# A randomised feasibility study to investigate the impact of education and the addition of prompts on the sedentary behaviour of office workers

**DOI:** 10.1186/s40814-017-0226-8

**Published:** 2018-01-15

**Authors:** Catriona O’Dolan, Margaret Grant, Maggie Lawrence, Philippa Dall

**Affiliations:** Institute for Applied Health Research, Glasgow Caledonian University, Cowcaddens Road, Glasgow, G4 OBA UK

**Keywords:** Sedentary behaviour, Prompts, Workplace, Office, Social cognitive theory

## Abstract

**Background:**

Office workers have been identified as being at risk of accumulating high amounts of sedentary time in prolonged events during work hours, which has been associated with increased risk of a number of long-term health conditions.

There is some evidence that providing advice to stand at regular intervals during the working day, and using computer-based prompts, can reduce sedentary behaviour in office workers. However, evidence of effectiveness, feasibility and acceptability for these types of intervention is currently limited.

**Methods:**

A 2-arm, parallel group, cluster-randomised feasibility trial to assess the acceptability of prompts to break up sedentary behaviour was conducted with office workers in a commercial bank (*n* = 21). Participants were assigned to an education only group (EG) or prompt and education group (PG). Both groups received education on reducing and breaking up sitting at work, and the PG also received hourly prompts, delivered by Microsoft Outlook over 10 weeks, reminding them to stand. Objective measurements of sedentary behaviour were made using activPAL monitors worn at three time points: baseline, in the last 2 weeks of the intervention period and 12 weeks after the intervention. Focus groups were conducted to explore the acceptability of the intervention and the motivations and barriers to changing sedentary behaviour.

**Results:**

Randomly generated, customised prompts, delivered by Microsoft Outlook, with messages about breaking up sitting, proved to be a feasible and acceptable way of delivering prompts to office workers. Participants in both groups reduced their sitting, but changes were not maintained at follow-up. The education session seemed to increase outcome expectations of the benefits of changing sedentary behaviour and promote self-regulation of behaviour in some participants. However, low self-efficacy and a desire to conform to cultural norms were barriers to changing behaviour.

**Conclusions:**

Prompts delivered by Microsoft Outlook were a feasible, low-cost way of prompting office workers to break up their sedentary behaviour, although further research is needed to determine whether this has an additional impact on sedentary behaviour, to education alone. The role of cultural norms, and promoting self-efficacy, should be considered in the design of future interventions.

**Trial registration:**

This study was registered retrospectively as a clinical trial on ClinicalTrials.gov (ID no. NCT02609282) on 23 March 2015.

## Background

High levels of sedentary behaviour (SB) have been associated with all-cause mortality [1] as well as contributing to the risk of developing a number of long-term health conditions including cancer, cardiovascular disease, diabetes, obesity, musculoskeletal problems, muscle degeneration, osteoporosis and depression [2–6]. This increased risk may be independent to the amount of physical activity an individual may perform [3, 7], and evidence suggests that prolonged and uninterrupted sedentary events have a greater negative impact on health than SB accumulated in shorter events [8].

Working-age adults are increasingly employed in low-activity occupations [9, 10], with office workers identified as one of the most sedentary occupational groups [11]. Office workers spend 65–75% of their working day sitting [12–16] and make significantly fewer breaks in sedentary time during working hours than in leisure time [9].

Targeting the SB of office workers has the potential to improve the health of individuals and positively impact presenteeism, absenteeism, and, ultimately, the economy [17]. A recent expert statement suggested that desk-based office workers should attempt to accumulate 2 h a day standing/light activity during working hours, eventually progressing to 4 h daily and take regular breaks from sitting [18]. However, a Cochrane Review noted that there was a lack of evidence from good quality studies of interventions that were effective at reducing SB in the workplace [19].

Providing office workers with sit-stand desks, facilitating continued use of a desk whilst standing, have demonstrated reductions in total sitting of approx. 60–90 min a day [19]. However, upgrading office furniture for an entire workforce may not be financially feasible, especially as the  long-term use and benefits to health of sit-stand desks is yet to be proven. Other studies have trialled one-to-one consultations with employees to initiate behaviour change, using tools such as mindfulness [20], behaviour change counselling [21] and person-centred consultation [22] to mixed effect. The cost implications of delivering such one-to-one interventions may also be an issue for large organisations.

The use of prompts as a way of reminding office workers to break up their sitting has had promising results [23–26]. Prompts, as a behaviour change technique, have been defined as an ‘environmental or social stimulus with the purpose of prompting or cueing the behavior’ [27] and may be useful at breaking the ‘habit’ of sitting [28]. Participants who received prompts achieved a significant reduction in the number (− 6.8%) and length (− 15.5%) of prolonged sitting events (> 30 min) [23], an increase in self-reported standing of + 7.99 ± 4.44 min a day [24], a significant reduction in total work day sitting (− 6.6%) and prolonged (> 30 min) events (− 13%) [25] and an increase in weekly work time standing of + 9% when prompts were delivered in conjunction with receiving a sit-stand desk [26]. Little was reported on the messages delivered by the prompts in these studies, but positively framed and tailored messages had been shown to have greater impact on health behaviours by enhancing individuals’ health intentions [29, 30].

As sedentary behaviour, as a distinct and independent health risk, is an emerging area of research, it cannot be assumed that all members of the general public are aware that sitting could be detrimental to their health. Providing such information usually forms part of interventions to reduce sitting, if not to intentionally form part of the behaviour change process, then to fulfil the ethical requirements of the research. However, this element is rarely described within SB interventions, let alone evaluated in terms of its impact. Where educational components are recorded, they are often too vague to draw comparisons or to elicit best practice. Yet, education could be important in forming individuals’ intentions to change their sedentary behaviour, and in doing so, form an essential foundation for subsequent components of an intervention to bring about action [31]. For example, prompts or the presence of a standing desk reminding people to act on the intention to break up sitting, initiated during an earlier education session.

There is a current lack of understanding with regard to how and why interventions such as prompts may be successful at reducing SB in a workplace setting. An important first step in gaining such understanding is identifying or developing explanatory theory for behaviour [32], which has so far been lacking in SB research [33]. Both the theory of planned behaviour [34] and dual process theory of motivation [31] have been studied with regard to explaining sedentary behaviour [35–39], but their inability to allow for the potential influence of the environment may prohibit them from fully explaining workplace SB. One theory that recognises the complex interaction between individuals and their environment is social cognitive theory (SCT) [40] which argues that the behaviour and opinions of those in our immediate environment are more likely to influence behaviour than regulations alone. SCT identifies five key influences for behaviour: (i) situation/environment, (ii) outcome expectations, (iii) self-efficacy, (iv) self-regulation and (v) observational learning. To date, SCT has not been applied to SB. This study aimed to investigate the feasibility and acceptability of using Microsoft Outlook as a vehicle for delivering customisable prompts to office workers, alongside education, to encourage breaks in SB during working hours. In addition, an indication of both the impact of, and behaviour change mechanisms behind, education and prompts on SB was sought through the collection of both quantitative outcome measures and qualitative data. It is recognised that whilst sample sizes may preclude definitive conclusions regarding efficacy, such conclusions may have important implications for future trial design. This study is structured using the updated CONSORT guidelines for reporting feasibility trials [41], and an adapted CONSORT flow diagram is presented.

## Methods

### Study design

This feasibility study was a 2-arm, parallel group and cluster-randomised trial. In which participants were allocated to one of the two clusters on a 1:1 basis. All aspects of the study were carried out onsite, at the participants’ place of work.

### Recruitment

A commercial UK bank involved in the Healthy Working Lives accreditation scheme was approached and agreed to take part in the study. Full- and part-time employees working in a large open-plan office were recruited via email and posters displayed in the workplace. The recruitment email was sent to all employees (~ 150). Volunteers were included if they met the following criteria: age 18 or over; self-reported that they were primarily engaged in sedentary, computer-based activities at a non-height adjustable desk during working hours; had access to Microsoft Outlook calendar; and absence of a pre-existing health condition that prohibited standing on a regular basis. All potential participants were given a participant information sheet and asked to sign a consent form.

### Education intervention

All participants attended an education session, delivered by a health-care professional, on the health risks associated with SB, the potential benefits of breaking up prolonged sitting, and tips on how to reduce sedentary behaviour at work. For example, prompting time management to facilitate regular breaks in sitting throughout the day. The information provided was based on recently published research in the field of occupational sedentary behaviour. Each education session lasted approximately 45–60 min.

### Randomisation

To minimise contamination between groups in the open plan office, participants were assigned to one of two equal sized clusters according to their geographical location in the office. Clusters were then randomly assigned to being in the Education Group (EG) who received no further intervention after the education session or the Prompt Group (PG) who received a 10-week prompt intervention in addition to the education session. Randomisation was achieved using sealed envelopes prepared by a third party. The researcher was blinded to cluster allocation during collection of activity data.

### Prompt intervention

Seventy brief, positively framed messages centred around taking breaks from sitting were compiled. Where appropriate, messages included the organisation’s name and location. For example, ‘Help Glasgow make a stand for better health!’ A custom Excel macro was used to assign one prompt message to a randomly generated time point every hour. Prompt times were restricted to a half hour period in the middle of each hour, to prevent prompts appearing close together, e.g. at 09.58 and again at 10.02. Prompts were generated for a period of 10 weeks, taking into account planned periods of absence from the office. A 10-week intervention period allowed adequate time for new behaviours to become a habit [42]. Microsoft Outlook was selected as a vehicle for delivering prompts to participants as it is widely available, has familiarity of use, poses no additional cost, and does not involve the security issues of downloading commercially available prompt software. Individual excel files containing prompt messages and timings were emailed to participants in the PG with instruction on how to upload the prompts to a newly created calendar in Microsoft Outlook. During the 10-week intervention period, prompts appeared as meeting reminders on the screen, which could be ‘snoozed’ or ‘dismissed’.

### Outcome measures

The primary outcome measures of the study were to assess the feasibility of the interventions in terms of:

- Eligibility, recruitment and follow-up rates.
- Acceptability of and utility of information from the education session.
- Acceptability and ease of use of prompts delivered by Microsoft Outlook.
- Insight into the experiences, motivations and barriers of participants with regard to making changes to SB.

These outcomes were assessed using logs kept of recruitment, retention rates and any operational issues regarding the prompts. Focus groups were conducted with participants from the EG and PG after a 12-week follow-up. A semi-structured focus group schedule was used to explore issues of acceptability of the interventions and gain insight into the experiences, motivations and barriers of participants with regard to making changes to SB. Focus groups were audio-recorded and transcribed verbatim, and two focus group moderators reviewed the transcripts for validation and accuracy.

The secondary outcome measures were to objectively measure changes in SB at three time points:

- Total sitting time: waking hours
- Total sitting time: work hours
- Number of sitting events per hour (work hours)
- Mean event duration of sitting events (work hours)
- Proportion of time spent in sitting events > 20 min (work hours)
- Proportion of time spent in sitting events > 30 min (work hours)

Sedentary behaviour was measured over 7 consecutive days using an activPAL3™ monitor (PAL technologies, UK) at baseline, 2 weeks before the end of the prompt intervention, and again at 12 weeks post-intervention. The activPAL is a tri-axial accelerometer worn on the midline of the anterior aspect of the thigh, and from the signal, time-stamps data into categories of sitting/lying, standing and walking. It has been validated as an accurate tool for capturing changes in posture and motion in adults during daily activities [43–45]. ActivPAL monitors were heat sealed inside transparent plastic tubing and further sealed with a waterproof dressing (Opsite Flexifix, Smith & Nephew). Participants were asked to keep a diary, recording waking and working hours, and any periods of non-wear, for the same 7-day period. Activity data was cross-validated with completed diaries. Data inconsistencies were logged, and where appropriate, data were excluded or non-wear time logged.

### Sample size

As this was a feasibility study, a sample size calculation was not performed. The study aimed to recruit 30 employees from a single workplace as a suitable sample to gather data on the feasibility and acceptability of the interventions. Data from key outcome measures were later used to determine the sample size for a definitive trial.

### Data analysis

Discussion regarding the acceptability of the prompt and education interventions was extracted from focus group transcripts. Thematic analysis was conducted on the remaining focus group data [46]. The broad themes which emerged from this process reflected key constructs of social cognitive theory (SCT) [40]. Therefore, the data was revisited, using the theoretical lens of SCT to finalise and refine the thematic analysis, to further insight into the experiences of the focus group participants.

Event-based outputs of SB from activPAL files were entered into customised Excel spreadsheets with wake and work times, and sedentary outcomes extracted. Events were classed as a continuous period of sitting with a start and end time [47]. The minimum data required for inclusion was 3 days of data, including at least one working day, for at least two of the three time periods. Inclusion criteria were set at this level in order to maximise the amount of data available to analyse from a small sample. SB outcomes were analysed using SPSS (Statistics Package for Social Sciences IBM version 22). Although this was a feasibility study, interferential statistics were performed to provide information about potential effectiveness. Differences between groups at all time points were compared using independent *t* tests, and Cohen’s *d* effect sizes were calculated. A post hoc sample size calculation was also performed.

## Results

### Eligibility, recruitment and retention

**Fig. 1:**
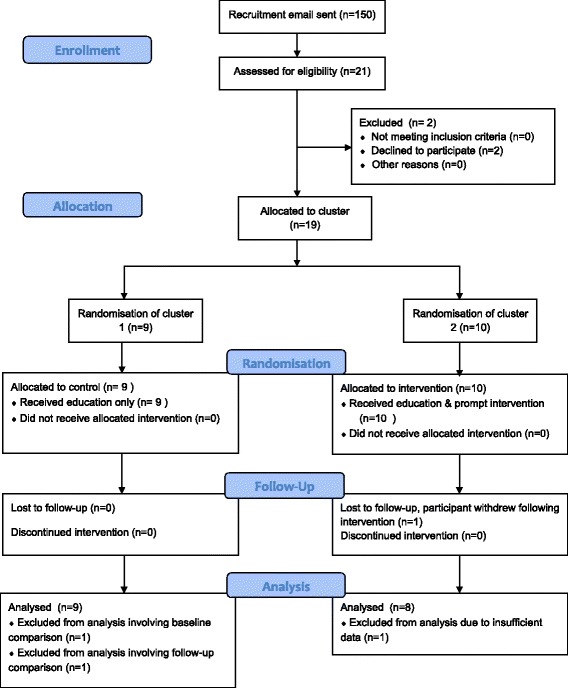
Adapted CONSORT flow diagram illustrating participant retention [41]

All those that volunteered to take part in the study satisfied the eligibility criteria, but the recruitment target of 30 participants was not achieved. Twenty-one participants were recruited (14% of those approached), and 86% remained in the study until the end (Fig. 1). Two participants withdrew prior to baseline measurement, and one after. One participant was excluded from analysis due to failure to meet minimum data collection requirements. Of the remaining 17 participants, 15 met the minimum data requirement for all three measurement periods. One participant met the requirements for the intervention and follow-up measurement periods only, and one participant met the requirement for baseline and intervention measurement only. Both these participants were in the EG. Due to the small sample size, these two participants were included in analysis where appropriate.

#### Demographics

The majority of the 17 participants were female (76%), aged between 22 and 56 years, employed full-time, and all self-reported their health to be at least good. Participants had a mean body mass index (BMI) of 25.1 kg/m^2^ which is categorised as ‘overweight’ [48], but individuals ranged from ‘healthy’ to ‘obese’ (BMI range 20.1–31.6 kg/m^2^) (Table 1). There was a gender difference between the EG and PG; there were no males in the PG but four (44%) in the EG. The PG also tended to be younger and more likely to rate their health as ‘excellent’ in comparison to the EG (Table 1). Participants were moderately active with a daily mean step count of 7751 (± 2038), with a mean of 2362 (± 1190) steps taken at work.

**Table 1:** Participant demographics at baseline

	Education group	Prompt group	Whole sample
Number of participants	9	8	17
Mean age [years]	42	36	39
Age range [years]	22–56	29–42	22–56
Male/female	44%/56%	0/100%	24%/76%
Self-reported general health			
Excellent	33%	62.5%	47%
Very good	33%	12.5%	24%
Good	33%	25%	29%
Mean BMI kg/m^2^ (SD)	25.6 (± 3.2)	24.5 (± 1.9)	25.1 (± 2.7)
Number of smokers	1	1	2
Employed full time/part time	67%/33%	75%/25%	71%/29%
Average daily step count	7617 (± 2041)	7886 (± 2167)	7751 (± 2038)
Average steps taken at work	2620 (± 1293)	2105 (± 1101)	2362 (± 1190)

The demographics of participants who took part in the focus groups were representative of the whole sample.

### Acceptability of education and prompt interventions

Focus group participants from both groups agreed that the education session had made them more conscious about their sitting behaviour and were shocked about the potential health consequences of too much sedentary behaviour. In particular, they were surprised that the risks were independent to the amount of physical activity undertaken.

The fact that I can sit all day and then go the gym at night, it still isn’t helping me.[PG2]

PG focus group participants spoke favourably about the content and variety of the messages delivered in the prompts.

I thought they were all good. Bit of information.[PG5]

However, focus group participants agreed that as time went on, prompts were less likely to be read or acted upon.

Eventually I think I switched off to them. I just saw the prompt coming up. Because I don’t have any other prompts so I knew what it was, and I didn’t even read it, I just…just missed it.[PG2]

No operational issues were logged regarding the uploading and operationalisation of prompts throughout the 10-week intervention period.

### Experiences, motivations and barriers for changing sedentary behaviour

Analysis of focus group transcripts identified five key themes which aligned with the main constructs of SCT: (i) situation/environment, (ii) outcome expectations, (iii) self-efficacy, (iv) self-regulation and (v) observational learning.

#### Situation/environment

This theme refers to the influence of the physical environment and context of sitting on SB. All participants felt that their sitting behaviour was linked to their physical environment and that they were far more sedentary in the office than at home in the evenings or on non-work days. The office environment was perceived to not be conducive to standing. There was a perception that work-related tasks could only be performed comfortably whilst sitting.

Our work is our work and we need to sit when we need to sit. It’s very difficult not to. [EG3]

The nature of the work being carried out did not lend itself to natural breaks in sitting, and it was noted by some that this had changed over the years;

Offices are trying to go paperless, it’s not like you’re getting up and going to the printer all of the time. [PG4]

Participants disagreed over whether changes to the physical office environment allowing workers to continue to work whilst in a standing posture, e.g. standing desks, would be agreeable. Some welcomed the opportunity to try such solutions, whilst others had tried a standing desk and did not like it.

I thought; ‘I can’t do this’ [and] sat back down again. [PG5]

#### Outcome expectations

Outcome expectations are the anticipation and value placed on the outcomes of reducing SB. Many participants were surprised by the health arguments for breaking up sitting presented to them during the education session. Although convinced by the health messages and their importance, without feedback to confirm their levels of SB, some were unsure if it applied to them.

I think I stand quite a lot because I always just print stuff and go and get it rather than wait. So… I don’t think prompts would really [work]. I would just probably get up before they prompted me. [EG2]

Participants in both groups spoke about experiencing some immediate benefits from taking breaks from their desks, although this was generally linked to being more mobile rather than just standing.

Actually getting up and walking away. Coming back you feel more refreshed than just standing up. [PG4]

#### Self-efficacy

Self-efficacy refers to ‘the belief in one’s capabilities to organize and execute the courses of action required to manage prospective situations.’ [49]. Many participants believed they did not have the ability to reduce the amount of time that they spent sitting at work. Sitting at work was reported as being ‘easy’ and taking breaks from sitting as ‘hard’. As well as the physical environment not facilitating standing, work was cited as the main barrier to breaking sitting. More than just the practical aspects of needing to be seated at a desk, work demanded concentration, leading to prolonged periods of SB occurring subconsciously.

Cos I do think I just don’t think about it, I just start [working] and it doesn’t even occur to me, I just get on with what’s in front of me, what I need to do. [EG5]

Whilst some spoke of a perceived lack of control over their SB, others admitted to purposefully waiting until lunch time to take a ‘comfort break’.

You’re like, ‘I might just wait [to go to the toilet] for lunchtime’. You do, you just sit there don’t you? [PG4]

Others felt that changing their SB at work was someone else’s responsibility.

We are the [education group] so we thought we wouldn’t do anything. [EG6]

The organisation should be more aware of it. [PG2]

A few individuals felt that they did have the ability to break up their SB and had taken steps to do so.

#### Self-regulation

Self-regulation refers to the behavioural strategies adopted by individuals in order to achieve the goal of reducing SB at work.

Participants from both groups spoke about strategies that they had used previously, or adopted since the start of the study, to reduce their SB. Many of these related to work tasks––designating specific tasks to be performed in non-sedentary postures.

I have changed. If I have got a lot of stuff to check, I’ll now go and stand by one of the high cabinets. I’ll stand instead of actually sitting at my desk. [EG1]

Breaking up SB was also health or emotionally led, with some participants reporting improved musculoskeletal problems, feeling more energised, and happier as a result of breaking up their sitting.

If I get 2 or 3 days where I’ve been in the office in a row I actually feel quite down. I deliberately organise my diary so that doesn’t happen. Just sitting at a desk all day, I’ve found I really struggle with that. [EG4]

Despite evaluating the content and variety of the prompt messages favourably, participants in the PG felt that they became less effective as time went on.

Eventually … Because I don’t have any other prompts so I knew what it was, I didn’t even read it, I just…just missed it. [PG5]

#### Observational learning

Observational learning refers to how individuals learned behaviour from observing others and how they believed their own behaviour was perceived. Participants in the EG said they had not noticed members of the PG standing, whilst those in the PG were very self-conscious about their changed behaviour. Often, derogatory terms were used to describe people acting out of the norm and standing or walking around the office.

People do look at you. If you’re just standing at your desk you look like an idiot. Most of the time I get up and try to get up and go somewhere, if you’re just standing there if you’re on the phone or something, people are just like [pulls a funny face] ‘what’s she doing?’ [PG5]

You wouldn’t make it that obvious [standing]. You would try and click [your mouse] or do something. Or people would think you’re a weirdo. [EG4]

Participants were therefore concerned about how their behaviour was perceived by others including, but not exclusively, management.

I think people would probably do it more if there was management buy-in because people are maybe not wanting to do it in case they’re [the management] not for it. [PG6]

A culture of conformity was described with the tendency for workers to eat lunch at their desks as this was the behaviour of the majority, i.e. the office ‘norm’.

You know if someone new moves in and sees everybody doing that [eating lunch at their desk] then they tend to drift towards that as well. [EG3]

Participants suggested that an intervention that everyone was participating in would be more acceptable. They felt that peer pressure and support would encourage breaks in SB within the office, as well as normalising the behaviour. This suggests that observational learning of SB is important, that workers learn and conform to the behaviour of the majority, and are concerned about how behaviour out with this norm is perceived by others.

### Outcome measures of sedentary behaviour

Data were found to be normally distributed for all outcome measures of SB at all three time points.

At baseline, participants (*n* = 16) spent on average 60.9 ± 5.9% (mean ± SD) of their waking hours (daily average 15.29 ± 1.08 h), in sedentary postures. During working hours (daily average 8.25 ± 0.99 h), this rose to 75.2 ± 17.4% of the time the monitor was worn at work, which equated to an average of 6.1 ± 1.5 h spent sitting at work a day. The mean duration of sitting events during work hours for the whole sample at baseline was 14.5 ± 5.7 min, with a mean of 3.5 ± 1.2 events per hour. Prolonged sitting in events of 20 min or more accounted for 49.4 ± 19.3% of time at work, and events of 30 min or more 36.0 ± 17.9% of time at work (Table 2).

**Table 2:** Sedentary behaviour outcomes for education only (EG) and prompt and education groups (PG) at three measurement points

Time point	Baseline		Intervention		Follow-up	
Group	EG (*n* = 8)	PG(*n* = 8)	EG (*n* = 9)	PG(*n* = 8)	EG (*n* = 8)	PG (*n* = 8)
Total sitting all days^a^ [%]	62.7 ± 8.9	60.9 ± 3.7	60.4 ± 9.0	62.3 ± 12.0	62.9 ± 12.3	60.1 ± 8.2
Difference between groups: mean (95% CI)	1.8 (− 5.5, 9.1)		− 1.9 (− 12.2, 9.0)		2.8 (− 8.4, 14.0)	
Effect size	0.264		− 0.180		0.268	
Total sitting work hours^b^ [%]	78.7 ± 11.8	71.8 ± 22.0	72.2 ± 15.0	69.4 ± 17.2	77.1 ± 11.7	70.0 ± 17.8
Difference between groups: mean (95% CI)	6.8 (− 12.7, 25.8)		2.8 (− 13.9, 19.4)		7.1 (− 9.0, 23.1)	
Effect size	0.387		0.172		0.472	
Sitting events per hour at work^b^ [number]	3.2 ± 1.1	3.8 ± 1.3	3.9 ± 1.5	4.2 ± 1.9	3.6 ± 1.5	4.0 ± 1.5
Difference between groups: mean (95% CI)	− 0.6 (−1.9, 0.63)		− 0.3 (− 2.0, 1.5)		− 0.4 (− 1.9, 1.2)	
Effect size	− 0.543		− 0.168		− 0.240	
Mean sitting event duration work hours^b^ [mins]	16.4 ± 5.2	12.5 ± 5.7	12.4 ± 4.9	11.6 ± 5.5	15.3 ± 7.9	12.6 ± 6.6
Difference between groups: mean (95% CI)	4.0 (− 1.9 2, 9.9)		0.9 (− 4.47, 6.2)		2.7 (− 5.11, 10.4)	
Effect size	0.722		0.168		0.368	
Time in event > 20 min work hours^b^ [%]	53.2 ± 15.0	45.6 ± 23.2	40.4 ± 21.0	39.6 ± 23.0	47.6 ± 23.0	43.0 ± 26.5
Difference between groups: mean (95% CI)	7.6 (− 13.4, 28.5)		0.8 (− 21.8, 23.5)		4.6 (22.1, 31.2)	
Effect size	0.389		0.039		0.184	
Time in event > 30 min work hours^b^ [%]	39.8 ± 13.4	32.2 ± 21.8	27.8 ± 20.0	28.0 ± 21.5	33.1 ± 23.6	33.6 ± 24.7
Difference between groups: mean (95% CI)	7.6 (− 11.8, 26.9)		− 0.2 (− 21.6, 21.2)		− 0.5 (26.4, 25.4)	
Effect size	0.419		− 0.008		− 0.021	

Data displayed in each cell are as follows: mean ± standard deviation were calculated using data on amount of time the activPAL was worn during waking hours^a^ and working hours^b^

Mean difference between groups is calculated as EG-PG (95% CI = 95% confidence intervals of the difference between group means), and effect sizes are calculated using Cohen’s d

*EG* education only group, *PG* prompt and education group

Comparison between the EG and PG for all key outcome measures showed a tendency for the PG to perform better in terms of a lower proportion of time spent sitting during work hours, less time spent sitting in prolonged (> 20 and > 30 min) events and more frequent events of shorter duration during working hours across all three time periods. The 95% confidence intervals of the difference between groups (independent *t* tests) were wide at all time points, including baseline, which may be due to the small number of participants, although effects sizes were mostly small < 0.2 or very small < 0.01 [50] (Table 2). Due to lack of differences at baseline, these variables were not controlled for in the analysis. A post hoc sample size calculation based on the data collected, estimated that a sample size of 27 participants per group would have been needed to show statistical differences between groups for the level observed.

There were small reductions from baseline to intervention in total sitting, sitting event duration and both measures of prolonged sitting during work hours in both groups, with the EG making greater reductions than the PG (% work hours sitting, EG = − 6.5%, PG = 2.4%; mean sitting event, EG = − 4 min, PG = − 0.9 min; time in events, > 20 min, EG = − 12.8%, PG = − 6%; time in events, > 30 min, EG = − 12%, PG = − 4.2%). These reductions were not maintained at follow-up (Table 2).

## Discussion

### Eligibility, recruitment and retention

The recruitment target of 30 participants was not met, despite an on-site study contact engaging with potential participants in person and by email. Future larger scale studies may need to consider recruiting across multiple work sites in order to attract larger numbers of participants. However, variations in worksite practices and occupational roles will need to be considered in terms of how these may influence results. Retention was good with 86% of participants remaining in the study until follow-up measurement.

### Acceptability of education and prompt intervention

The content of the education sessions and prompts were evaluated favourably by focus group participants. The process of generating and uploading prompts proved straightforward, with no operational issues reported. In this regard, using Excel and Microsoft Outlook proved to be a feasible, low-cost method of providing office workers with randomly timed reminders to break up their sitting. However, despite variation between 50 prompt messages, participants reported ignoring prompts before the end of the intervention period, and with lack of evidence that prompts resulted in greater reductions in SB than education alone, further research is needed to ascertain whether it is worthwhile rolling out this intervention on a larger scale.

### Experiences, motivations and barriers for changing sedentary behaviour

Thematic analysis of focus group data identified themes in line with the key constructs of social cognitive theory [40]. This has provided valuable insight into why changes were or were not made to SB during and after the intervention.

Focus group participants were in agreement that both the office environment and the situation of performing work tasks greatly influenced their SB at work. Analysis of the transcripts suggested that the education session was successful at increasing participants outcome expectations regarding the benefits of changing their SB. Studies have shown that individuals are more likely to act on health issues if they perceive they are susceptible to the problem; it has serious consequences and that a course of action will minimise these consequences [51]. However, not all participants were convinced that they needed to improve their SB patterns, suggesting that providing feedback on baseline SB may be valuable in terms of encouraging change and setting behaviour goals. Goal setting and feedback are common behavioural strategies associated with self-regulation of behaviour [52]. A recent review of techniques used in SB interventions found that setting behavioural goals was the most frequently discussed behavioural change technique [53].

Following the education session, some participants that developed their own behavioural strategies to break up their sitting had identified specific work tasks to be done whilst standing. This method not only provided an existing cue (the task) for changing posture but also fulfilled the desire to be carrying out a work task whilst standing that was expressed by participants in both groups. In addition, by associating particular tasks with standing, some of the need to make a conscious decision to stand was removed. Evidence suggests that when a behaviour is not demanding and more easily engaged in (e.g. sitting at a desk), cognitive control systems give way to regulation by lower control systems in which behaviour is automatic and less consciously thought about [40, 54]. Assigning tasks to standing could therefore be an effective strategy that has the potential to result in long-term behaviour change.

Focus group discussions suggested that an important barrier to changing SB was low self-efficacy. Participants felt that they did not have the ability to change their SB during working hours due to be the pressure of work tasks that prevented taking a break or caused them to lose track of time. This endorses the findings of other studies in which participants cited perceived time pressures [55] and interruptions to productivity [56] as barriers to taking breaks from sitting. In this way, employees felt they were not to ‘blame’ for their sitting behaviour, almost absolving themselves from responsibility, an attitude at odds with the UK NHS manifesto to empower citizens with greater control over their health and care [57].

Observational learning or how non-sitting behaviour was perceived by and replicated (or not) by others was important to focus group participants. They perceived that they would look ‘weird’ and ‘strange’ if they stood at their desks and expressed a desire not to be carrying out a behaviour that was not the office norm. Changing what constitutes normal behaviour within an office is likely to be the key to facilitating large-scale behavioural change to reduce sitting at work. It will involve changing the culture not only in terms of behaviour, but in terms of the environment, policies, leadership and individual beliefs. Little is understood about how to facilitate such change. The focus of studies on workplace culture tends to centre around improving employee performance and productivity [58, 59] rather than health. If breaking SB at work is seen to negatively correlate with the global objective of improved productivity, then this will pose a serious barrier to encouraging changes in work practice. Employers, perhaps, need to be convinced of the health benefits of reducing SB in their employees in terms of gains in productivity and reduced losses due to sickness absence.

Focus group participants spoke about the study having increased their knowledge and outcome expectations, and, for some, their ability to self-regulate their SB. However, low self-efficacy and a desire to conform to normal sitting behaviour proved to be barriers to change. Health promotion interventions which utilise social support and increase self-efficacy are more likely to have a positive outcome [60]. Future interventions to reduce SB in the workplace should look at ways of focusing on these constructs of SCT in order to maximise behaviour change.

### Outcome measures of sedentary behaviour

Both the education and prompt group had a reduction in mean from baseline to intervention measurement for total sitting, sitting event duration and both measures of prolonged sitting during work hours. Evidence from focus group discussions suggest this decrease may have been the result of the education sessions, with participants from both groups adopting their own behavioural strategies to break up SB. Focus group participants felt that the prompts had been initially effective, but the impact had reduced during the intervention; it could be that the timing of the second measurement period was too late to capture such short-term changes. A significant reduction in the number and length of prolonged sitting events in a prompt intervention group has been demonstrated in the first 5 days of prompt delivery [23]. Future studies should attempt to clarify if there is a short-term impact of prompts on SB and at what point they begin to lose effectiveness in order to modify intervention design to facilitate longer term behaviour change.

### Limitations

The recruitment target for this study was not met, and ways of maximising recruitment should be considered in future studies. Although not its primary aim, the small sample size did not allow inferences to be drawn regarding the statistical significance of the differences in SB outcomes observed. Based on our post hoc calculations, a definitive trial should seek to recruit a minimum of 27 participants per group in order to be able to detect significant changes in SB outcomes. Whilst cluster randomisation was used to minimise contamination between groups, it also means that what might be being measured is behavioural patterns influenced by neighbouring colleagues rather than solely the impact of the intervention. Non-clustered randomisation to groups might have prevented such influences, but at the same time, it is important to acknowledge that participants felt self-conscious of behaviour not perceived to be the norm by others in the office. Participants’ baseline measurements may have been influenced by information about SB in the UK media occurring around the same time, and also the information given to them in the participant information sheet, explaining the purpose of the study. Either or both of these factors may have led them to alter their SB during baseline measurement. Whilst it is not possible to eliminate exposure to media, steps could be taken to minimise how much information was given in the study materials, e.g. participant information sheet. Blinding participants to the study hypothesis has been suggested as a method of reducing bias in trials on non-pharmacological interventions [61].

## Conclusions

This feasibility study provides a valuable contribution to research into the use of prompts and education on changing the SB of office workers. Focus group participants evaluated both of these components favourably, and they provide a low-cost solution that would be easy to implement on a wider scale. The education component seemed to be successful at increasing outcome expectations and self-regulation in some individuals, leading to short-term reductions in SB. However, further research is needed to determine whether there is any added benefit to providing prompts in addition to education, and how any short-term behaviour change can be translated into long-term changes that are sustainable over time. The role of office culture in terms of what is seen as normal behaviour, and ways of increasing self-efficacy may play an important part in future intervention design.

## Abbreviations

EG: Education only group
PG: Prompt and education group
SB: Sedentary behaviour
SCT: Social cognitive theory
